# *Notes from the Field:* An Outbreak of Shiga Toxin–Producing *Escherichia coli* O121 Infections Associated with Flour — Canada, 2016–2017

**DOI:** 10.15585/mmwr.mm6626a6

**Published:** 2017-07-07

**Authors:** Vanessa Morton, Joyce M. Cheng, Davendra Sharma, Ashley Kearney

**Affiliations:** ^1^Centre for Foodborne, Environmental, and Zoonotic Infectious Diseases, Public Health Agency of Canada; ^2^Office of Food Safety and Recall, Canadian Food Inspection Agency; ^3^National Microbiology Laboratory, Public Health Agency of Canada.

On December 29, 2016, PulseNet Canada identified a cluster of six *Escherichia coli* non-O157 isolates with a matching pulsed-field gel electrophoresis (PFGE) pattern combination that was new to the PulseNet Canada database. The patients resided in three geographically distinct provinces. In January 2017, the Public Health Agency of Canada (PHAC) initiated an investigation with local, provincial, and federal partners to investigate the source of the outbreak.

A case was defined as isolation of *E. coli* non-O157 with the outbreak PFGE pattern or closely related by whole genome sequencing (WGS) in a Canadian resident or visitor with onset of symptoms of gastroenteritis on or after November 1, 2016. Patients’ illness onset dates ranged from November 2016 to April 2017 (Figure). As of May 23, 2017, a total of 29 cases were identified in six provinces (Alberta, British Columbia, Newfoundland and Labrador, Ontario, Quebec, and Saskatchewan). One additional case was identified in a U.S. resident who traveled to Canada during the exposure period. Patients’ ages ranged from 2–79 years (median = 23.5 years) and 50% were female. Eight patients were hospitalized, and one developed hemolytic uremic syndrome. Clinical isolates were typed as *E. coli* O121:H19 (one case was typed as *E. coli* O121:H undetermined) with Shiga toxin 2–producing genes by in silico toxin testing and had closely related PFGE patterns and WGS.

**FIGURE:**
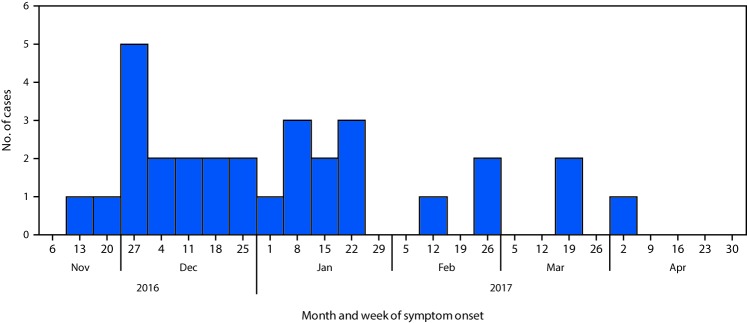
Number of confirmed cases of *Escherichia coli* O121 infection (n = 30),* by week of symptom onset — Canada, November 2016–April 2017 * One case occurred in a U.S. resident who traveled to Canada during the exposure period.

Initial investigation into the source of the outbreak did not identify any clear hypotheses; common exposures were ground beef, sausage style deli-meats, pizza, and pork, but the data did not converge on any specific products. Patients were reinterviewed by PHAC using an open-ended approach. Knowledge of a recent *E. coli* O121 flour-associated outbreak prompted interviewers to ask about baking and exposure to raw flour or dough (*1*). Patients were also asked if any food items of interest, including flour, were available for testing.

In March 2017, *E. coli* O121 with the outbreak PFGE pattern was isolated from an open flour sample from a patient’s home and a closed sample collected at a retail store, both of the same brand and production date. The clinical and flour isolates grouped together, with only 0–6 whole genome multilocus sequence typing allele differences. As a result of these findings, a product recall was issued. Based on possible connections to the recalled lot of flour, market sampling of flour within certain periods was initiated. The investigation led to additional recalls of flour and many secondary products (*2*).

As of May 23, 2017, 22 patients had been asked about flour exposure in the 7 days before illness onset; 16 (73%) reported that the implicated brand of flour was used or probably used in the home during the exposure period. Comparison data on the expected proportion with exposure to this brand of flour were not available. Eleven of these sixteen patients reported they ate or probably ate raw dough during their exposure period.

This is the first national outbreak of non-O157 Shiga toxin–producing *E. coli* infections identified in Canada and the first Canadian outbreak linked to flour. An open-ended interview approach and flour sampling were used to implicate flour as the source. Because of the recent emergence of *E. coli* outbreaks linked to flour, public health professionals should consider flour as a possible source in *E. coli* outbreaks and communicate the risk associated with exposure to flour, raw batter, and dough in public health messaging.
